# Account of Diseases in an Eastern District of London, from the 20th of December, 1800, to the 20th of January, 1801

**Published:** 1801-02

**Authors:** 


### *Account of Diſeaſes in an Eaſtern Diſtrict of London, from the 20th of December, 1800, to the 20th of January, 1801.*

| | No. of Caſes. |
|---|---|
| ACUTE DISEASES. | |
| Continued Fever | 18 |
| Pneumonia | 2 |
| Sore Throat | 3 |
| Acute Rheumatiſm | 2 |
| Dyſentery | 2 |
| CHRONIC DISEASES. | |
| Cough | 15 |
| Cough with Dyſpnœa | 19 |
| Hydrothorax | 4 |
| Catarrh | 5 |
| Hæmoptyſis | 4 |
| Gaſtrodynia | 10 |
| Aſcites | 3 |
| Anaſarca | 5 |
| Colica | 2 |
| Enterodynia | 5 |
| Hyſteria | 7 |
| Vertigo | 4 |
| Hemiplegia | 2 |
| Cephalæa | 8 |
| Amenorrhœa | 9 |
| Fluor Albus | 7 |
| Hæmorrhois | 3 |
| Dyſuria | 2 |
| Chronic Eruptions | 12 |
| Lumbago | 2 |
| Sciatica | 2 |
| Chronic Rheumatiſm | 19 |

PUERPERAL

| PUERPERAL DISEASES. | | INFANTILE DISEASES. | |
|---|---|---|---|
| Menorrhagia Lochialis - | 5 | Aphthæ - - - - - - - | 4 |
| Low Puerperal Fever - - | 4 | Convulſio - - - - - - | 3 |
| Rhagas Papillæ - - - - | 3 | Diarrhœa - - - - - - | 8 |
| | | Dentition - - - - - - | 2 |

We are ſorry to announce the continuance of a diſeaſe, which we have in ſeveral former Reports, either particularly deſcribed, or incidentally referred to, as the reigning epidemic: A continued fever of the typhoid character, and attended with unuſually ſevere affections of the head, ſtill prevails very generally.

The delirium which commonly attends fevers of this claſs, is rather a confuſion of ideas and an interrupted recollection, attended with low mutterings and expreſſions of anxiety, than any diſcovery of rage and violence; but in many of the caſes now referred to, there has been an active and furious delirium, which has rendered coercive means neceſſary to keep the patient in bed, or prevent him from doing miſchief. The diſeaſe has in ſome inſtances approached in a very inſidious manner, and before the ſtate of the pulſe and other ſymptoms have created any alarm, either coma or delirium have occurred, and excited apprehenſions for the ſafety of the patient; and in too many inſtances the diſeaſe has proved fatal.

In a late report, we had occaſion to remark that the Small-pox proved uncommonly fatal; and by a compariſon of the liſts now referred to, it will appear that the number of deaths by this diſeaſe during the laſt year, is in the proportion of two to one of the preceding year. The number of deaths by Fever, in the ſame period, has alſo increaſed by more than one-third. *

* *The General Bill, containing the Diseases and Caſualties of the year* 1800, *is unavoidably poſtponed till our next.*

